# Hypertrichotic Giant Nevus Spilus Tardivus and Neurofibroma of the Tongue in Sporadic von Recklinghausen's Disease

**DOI:** 10.1155/2014/141075

**Published:** 2014-11-11

**Authors:** Prabhath Ramakrishnan, Vijay Sylvester, Prathima Sreenivasan, Janisha Vengalath, Smruthi Valambath

**Affiliations:** ^1^Department of Oral Medicine and Radiology, Kannur Dental College, Anjarakandy, Kannur, Kerala 670612, India; ^2^Department of Physiology, SDM College of Medical Sciences, Dharwad, Karnataka 580009, India

## Abstract

Solitary neurofibromas are rare, benign tumours of nonodontogenic origin. The presentation of a solitary neurofibroma on the tongue is an uncommon occurrence and we present such a case here which was discovered in concomitance with multiple neurofibromatosis type 1 (von Recklinghausen's disease). Such a rare presentation seen in this case is a diagnostic challenge and often clinched only with the aid of histopathological and immunohistochemical examination. This work also discusses the various differential diagnoses that can be considered in similar cases. The presence of a hypertrichotic “giant” nevus spilus tardivus (Becker's nevus) is also a rare finding in this particular case. We present such a case which will be of interest to the budding dental practitioner. The lesion was excised and the patient followed up without any evidence of malignant transformation.

## 1. Introduction

Neurofibromas are benign nerve sheath tumors originating from the peripheral nerves and are the hallmark presentation in von Recklinghausen's disease or neurofibromatosis type 1 [1].

Neurofibromatosis is a rare disease that includes two variants that is divided into neurofibromatosis type 1 (NF1) and type 2 (NF2). NF1 is the most common of these two types with a frequency of 90%, compared with 10% for NF2. Among these the NF1 is also called the peripheral neurofibromatosis, popularly called von Recklinghausen's disease with a reported prevalence of 1 : 5000 in the population and a birth incidence of 1 in 2500–3300 [2]. There are clinical criteria suggested by the National Institute of Health (NIH) Consensus Development Conference to classify a patient as having NF1. The patient has to have 6 or more café au lait spots equal to or larger than 0.5 cm in prepubertal individuals and equal to or larger than 1.5 cm in postpubertal individuals; 2 or more neurofibromas of any type or 1 or more plexiform neurofibroma; inguinal or axillary freckling (Crowe's sign); an optical nerve glioma; 2 or more benign iris hamartomas (or Lisch nodules); a distinctive osseous lesion: dysplasia of the sphenoid bone, dysplasia or thinning of long bone cortex with or without pseudarthrosis, and a first degree relative with NF1 [3–5].

Neurofibromatosis is an autosomal dominant disorder and sporadic incidences are not very uncommon (30–50%). The genetic mutation is located on the NF1 gene and can be traced to chromosome 17q11.2. Gene mutations usually cause a deficiency of a tumour suppressor protein product called neurofibromin. So, there is uncontrolled cell proliferation that results in the growth of a tumour. The penetrance of NF1 gene is 100% in adults, but, there is a high variance in expression [5]. The neurofibromas in the oral cavity most commonly involve the tongue, followed by lips, palate, buccal mucosa, gingiva, floor of the mouth, or the pharynx [6].

Although there are numerous reports of the incidence of plexiform neurofibromas involving the oral cavity and head and neck regions, solitary plexiform neurofibromas of the tongue are rare lesions and one does not usually come across them in day to day practice; however, in most cases their coexistence with von Recklinghausen's disease has been quite well established as seen from reports around the world. A search of the reported cases in the past 15 years was conducted and to the best of our knowledge this is the very first case of a plexiform neurofibroma of the tongue reported in a male patient in literature (Table 1) [7–13].

The differentiation between neurofibroma and the plexiform variety is usually clinched with histopathological and immunohistochemical diagnosis. This case report presents with a solitary plexiform neurofibroma involving the tongue which is not a very common presentation with sporadic neurofibromatosis type 1. It is also interesting because the case presents with a hypertrichotic giant nevus spilus tardivus in von Recklinghausen's disease involving the right arm which has very rarely been reported in literature.

## 2. Case Presentation

A 39-year-old Asian male presented to our department with a chief complaint of a painless swelling involving the left lateral border of the tongue. History revealed that the growth began as a pea-sized nodule and gradually increased in size to its present state over a period of 4 months.

**Figure 1:**
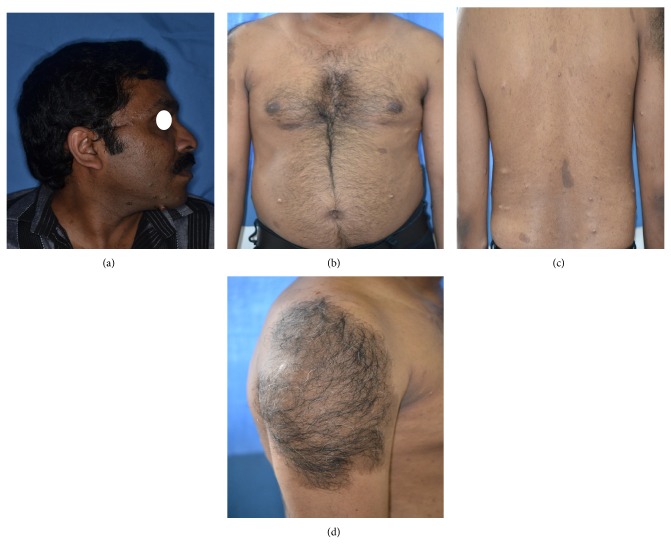
(a) Right profile view revealing multiple cutaneous nodules. (b) Multiple cutaneous nodules and café au lait macules on the trunk. (c) Multiple cutaneous nodules and café au lait macules on the back. (d) Hypertrichotic giant café au lait macule on the right arm.

The past medical history did not reveal any cardiovascular, respiratory, genitourinary, gastrointestinal, endocrine, haematological, neurological, or any other medical history of relevance. The past dental or familial history was not of any consequence to our particular case. A complete blood count was performed and no abnormality was detected. No history of trauma, bleeding, pain, or paresthesia was present. There were no signs of any cervical lymphadenopathy noted. Orthopantomographic examination did not reveal any bony abnormalities. Extraoral examination revealed multiple soft cutaneous nodules involving either side of the face, back, trunk, and the lower and upper extremities. They were round to oval in shape and were of various sizes ranging from a few millimetres to centimetres across. On palpation they were found to be sessile and some pedunculated also, soft to firm in consistency, nontender, and noncompressible and showed no signs of fixity. Around 15 café au lait (coffee in milk) macules were present over 15 mm in diameter throughout the body with increased prevalence in the back and trunk. The largest one among these was located over the right arm measuring a whopping 22 × 13 cm across with smooth borders. It was roughly ovoid in shape and there was a brownish macule with long thick dark hair involving the surface of the lesion and it extended from the acromioclavicular joint all the way down to the upper arm (Figure 1). The distribution of the lesion did not follow the lines of Blaschko. Rubbing the affected area exhibited a pseudo-Darier sign which consisted of a transient piloerection. Neither axillary freckling (Crowe's sign) nor Lisch nodules were noted in our case.

**Figure 2:**
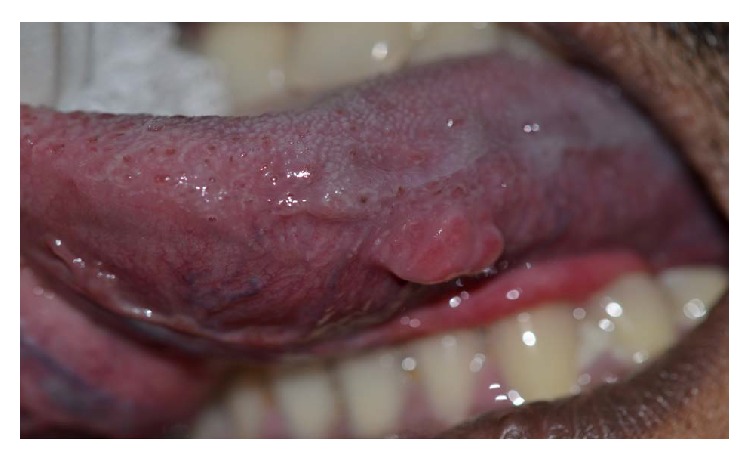
Nodular growth on the left lateral border of tongue.

Intraoral examination revealed a sessile lesion with a lobulated appearance and measured 2 × 1.5 cm in greatest dimensions. It was roughly ovoid in shape with irregular borders and exhibited a smooth surface. The periphery was non-erythematous in appearance. On palpation it was found to be nontender, firm in consistency, and fixed to the underlying tissue. It was noncompressible, nonreducible, and nonpulsatile in nature (Figure 2). The slow-growing, asymptomatic nature of the lesion with the presence of well circumscribed margins led us to give a provisional diagnosis of a benign lesion. Based on the positive clinical features in our extra- and intraoral assessment of the patient, a provisional diagnosis of a neurofibroma was considered, taking into consideration the fact that the patient revealed pathognomonic signs of neurofibromatosis type 1 extraorally.

Considering the clinical presentation and localization of the lesion, we included a neurofibroma, schwannoma, neurilemmoma, granular cell tumour, reactive lesions like a giant cell fibroma or focal fibrous hyperplasia, leiomyoma, rhabdomyoma, hemangioma, lymphangioma, lipoma, and benign salivary gland tumours among differential diagnosis taking into account the peripheral exophytic nature of the lesion.

Routine haematological examination revealed a normal blood profile and no other imaging findings for soft tissue analysis like ultrasonography or MRI were performed considering the miniscule proportions of the lesion and its benign presentation. Patient was also referred to the adjacent medical college to rule out the possibility of any internal lesions. Excisional biopsy of the tumour mass on the tongue was performed in toto and primary closure achieved with a single interrupted Vicryl suture. The patient was prescribed an NSAID medication and asked to use povidone-iodine mouth rinse.

**Figure 3:**
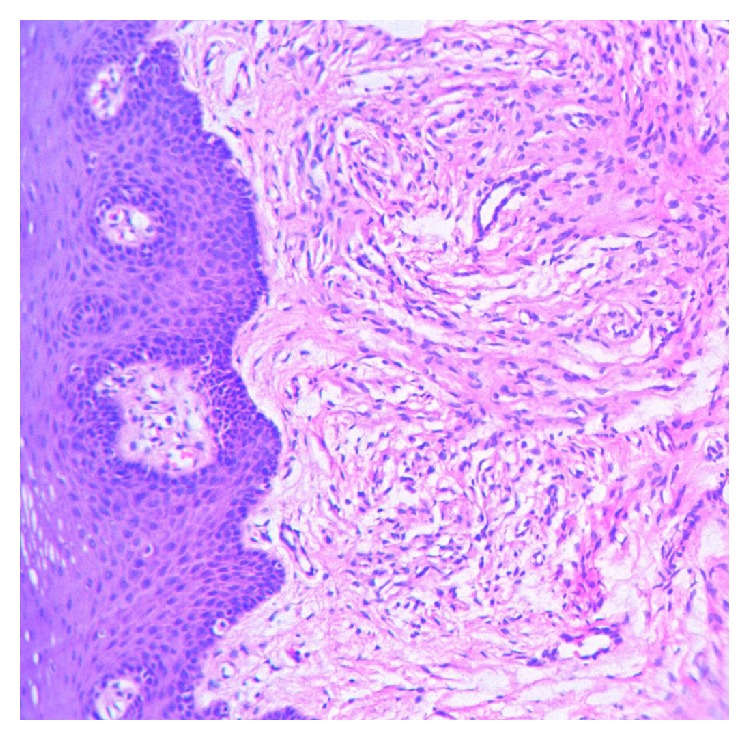
Photomicrograph revealing spindle cells as fascicles with thin wavy nuclei.

**Figure 4:**
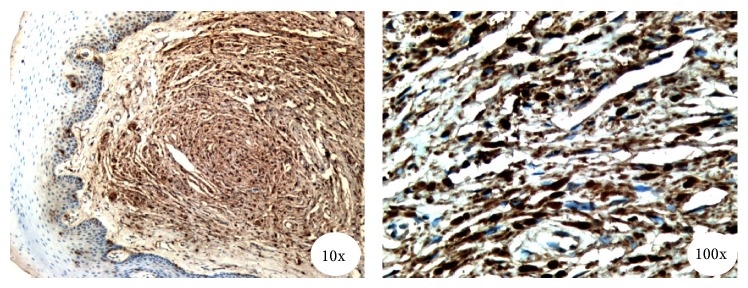
Photomicrograph exhibiting immunoreactivity for S-100 protein in spindle cells. (10x) and most of the tumour cells are positive for S-100 protein (100x).

**Figure 5:**
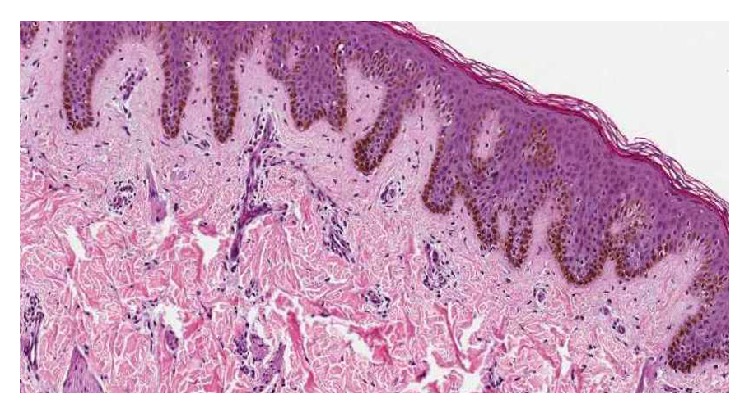
Photomicrograph reveals elongated rete ridges and pigmentation of the basal layer with no increase in the number of melanocytes.

Histopathological examination which is the investigation of choice in peripheral exophytic lesions involving the tongue revealed a section with densely collagenous stroma with proliferation of spindle cells as fascicles with thin wavy nuclei in irregular pattern. Numerous plump fibroblasts were present and there were many vascular channels. Connective tissue is lined by stratified squamous epithelium (Figure 3). The specimen further underwent immunohistochemical analysis, which can be considered a gold standard, and was found to be immunoreactive for S-100 stain which was positive for the spindle cells, thereby, signifying its origin from neural crest tissue and confirming our diagnosis of a neurofibroma (Figure 4). The lesion on the shoulder underwent an incisional biopsy and on histopathological examination revealed numerous elongated rete ridges with melanin pigmentation of the basal layer with no increase in the number of melanocytes. The specimen tested negative for S-100 protein (Figure 5).

The postoperative healing was uneventful and patient was followed up after two weeks and subsequently after 6 months. The patient was also advised to report for periodic follow-up visits due to the potential for neurofibromas to undergo malignant transformation.

## 3. Discussion

A neurofibroma is a benign tumour arising from the cells of neural sheath origin. Although an uncommon benign tumour, we found it prudent to place the diagnosis of a neurofibroma with a higher ranking in our list of differential diagnoses considering the fact that our patient exhibited other extraoral features of neurofibromatosis type 1. It usually presents on the tongue and also occasionally on the buccal mucosa, gingiva and involves the lips very rarely [1–3]. The expression of the condition shows a wide variation. Some individuals may have thousands of neurofibromas on the body whereas some may have few like our patient. The neurofibromas involving the tongue are almost always nodular in nature as was present in our case. The patient presented to us with a complaint of a slow growing mass involving the left lateral border of the tongue. The patient was educated and aware about the condition developing on his tongue and wanted expert consultation. It is important that the general public is made aware regarding the importance of having such lumps and swellings examined by a clinician and the possibility of malignancy ruled out. The differential diagnosis of enlarging tongue masses includes schwannoma, granular cell tumour, reactive lesions like a giant cell fibroma or focal fibrous hyperplasia, leiomyoma, rhabdomyoma, hemangioma, lymphangioma, lipoma, and benign salivary gland tumours among differential diagnosis taking into account the peripheral exophytic nature of the lesion.

A neurilemmoma or a schwannoma is an encapsulated tumour mass that is present submucosally. They are not very common in the oral cavity, but if present, the tongue is the most common location for the lesion. They appear nodular and sometimes may grow to fantastic sizes. Confirmatory diagnosis is by conducting a biopsy [14].

Granular cell tumour, also called the Abrikossoff's, may occur anywhere in the body but has a marked predilection for the tongue when it occurs in the oral cavity. Usually it presents in the fourth to sixth decades of life. In over 50% of the cases tongue shows pseudoepitheliomatous hyperplasia, but, IHC can help in diagnosis with the aid of S-100 immunoreactivity in the mesenchymal tissues [15].

Another reactive lesion, namely, the focal fibrous hyperplasia (irritation fibroma), typically presents as a smooth nodule. It is usually 1.5 cm or smaller in diameter. Prevalence is greater in individuals above 40 yrs of age [16].

Oral leiomyomas are benign smooth muscle tumours that are rarely noticed in the oral cavity and most of them, if present, arise from the smooth muscles of the underlying vasculature. They generally present as tiny, slow growing, solitary nodular masses located principally on the tongue, lips, palate, and buccal mucosa. Although asymptomatic, they may present with symptoms such as pain, tooth mobility, or difficulty in chewing [17, 18].

Rhabdomyomas are striated muscle tumours which may occur on the mucosal surfaces as well. They are usually very rare and diagnosis can be confirmed by normal H&E staining. However, they have to be biopsied to evaluate the presence of its extremely malignant counterpart, the rhabdomyosarcoma [19].

Hemangioma of the tongue is highly unusual occurrences and is more common in the 1st decade of life. Intramuscular tumours are nonmetastasizing benign congenital tumours. Most of them also increase in size in 2nd-3rd decades of life. They are usually not life threatening entities [20].

Lymphangioma is a benign proliferation of lymphatic vessels and is often hamartomatous transformations of malformed lymphatics. They may present as localised growths and their limited extension facilitates easy surgical removal. They are difficult to diagnose based on the clinical appearance alone and are mainly caused because of the difficulty in drainage of the lymph which causes a localised collection in the area [21].

Lipoma is a painless benign mesenchymal tumor that is well circumscribed. It may be present in any site but is rare in the oral cavity. Possible causes include infection, chronic irritation, hormonal imbalance, and trauma. Histopathological analysis is the gold standard for diagnosis and they are composed of mature far cells with fibrous connective tissue which is often hyalinised with or without the capsule and/or fibrous septa [22].

The majority of salivary gland tumours are benign in nature, with pleomorphic adenoma accounting for 60% of them. The involvement of the tongue is very rare for a pleomorphic adenoma and is hence ranked the last [23, 24].

There is still some controversy regarding the presence of Lisch nodules associated with neurofibromatosis type 1, which was not present in our case, which certain authors think are asymptomatic and not correlated with visual impairment or severe optic nerve involvement [25]. Our case also revealed a giant nevus spilus tardivus macule. They histologically reveal increased melanin deposition by the underlying basal keratinocytes and the melanocytes [26]. The lesion on his tongue was excised under local anaesthesia. An incisional biopsy of the lesion on his shoulder was performed and subjected to histopathological examination which revealed several elongated rete ridges with pigmentation of the basal layer. To the best of our knowledge hypertrichosis involving a giant café au lait macule has never before been reported in literature and is a novel finding in our case. The café au lait spots produced in neurofibromatosis type 1 are individually indistinguishable from nevus spilus or in this case with the overlying hypertrichosis. In fact when multiple nevus spilus is seen, there is a possibility of café au lait spots of neurofibromatosis type 1. They can be ideally differentiated only by performing immunohistochemical analysis for S-100 protein [27]. In our case, the sample from the shoulder tested negative for the S-100 protein. Although it has been proven that café au lait spots are associated with von Recklinghausen's disease, we require more case reports with immunohistochemical analysis to associate these kinds of hypertrichotic giant nevus spilus with NF1. In this case, the patient revealed that he remembers the nevus was not congenital, appearing at the age of 12, and gradually grew in size and became hypertrichotic over a period of 5 years.

As was in our case, most of the time, neurofibromas are usually not with significant clinical consequences; a feared complication of neurofibromas is the probability for malignant transformation which is the reason why it is most prudent to perform biopsies of the tumour masses, as we have done. The incidence of sarcomatous transformation has been placed at 15% of all cases by Preston and coworkers. Fibrosarcoma, spindle cell sarcoma, and neurogenic sarcoma are some of these. In addition, Preston and coworkers have also mentioned other pathological lesions, mental disorders, osseous defects, and congenital defects which were not seen in our case [28]. In the specimen we excised, we obtained immunohistochemical analysis for S-100 protein, which was adequate for the diagnosis of neurofibromas. However, if they do not show positive immunoreactivity or if there is difficulty in differentiation between different neural tumours we can apply other staining tests like epithelial membrane antigen (EMA), factor XIIIa, CD34 or CD68, or type IV collagen [29]. Since our patient is showing other signs of multiple neurofibromatosis regular follow-up visits are required to detect any features of malignant transformation. Rarely, cases like this present us with a unique opportunity to diagnose neurofibromatosis. Although the oral manifestations of von Recklinghausen's disease are well documented, it may not be at the forefront of the inexperienced clinician's mind while diagnosing nodules of the tongue. Extraoral examination of all the patients who visit the dental operatory is of paramount importance as this case suggests, for one may miss the “bigger” picture if we as dental practitioners concentrated only on the oral findings of a particular condition. It is also important considering the fact that neurofibromas have a rare malignant transformation potential.

## Figures and Tables

**Table 1:** Isolated plexiform neurofibromas of the tongue case reports over the past 15 years [7–13].

Location	Age/sex	Year	Authors
Tongue	39/M	2014	Present case
Tongue	11/F	2013	Sharma et al. [7]
Tongue	34/F	2012	Iyer et al. [8]
Tongue	5/F	2010	Sirinoglu et al. [9]
Tongue	3/F	2006	Guneri et al. [10]
Tongue	24/F	2006	Marocchio et al. [11]
Tongue	35/F	2006	Bongiorno et al. [12]
Tongue	5/F	2006	Guclu et al. [13]
